# Common and Divergent Physiological Responses to Hypoxia and Heat

**DOI:** 10.1007/s40279-026-02419-2

**Published:** 2026-05-13

**Authors:** Yohan Rousse, Franck Brocherie, Grégoire P. Millet

**Affiliations:** 1 Laboratory Sport, Expertise and Performance (EA 7370), French Institute of Sport (INSEP), Paris, France; 2 University Paris Cité, Paris, France; 3 Institute of Sport Sciences, Faculty of Biology and Medicine, University of Lausanne, 1015 Lausanne, Switzerland

## Abstract

Exposure to environmental stressors such as hypoxia or heat has emerged as an effective strategy to stimulate physiological adaptations, enhance sport/physical performance and promote health. Both environmental stressors activate shared molecular pathways, notably through the stabilisation of hypoxia-inducible factor-1 alpha and the induction of heat shock proteins, which mediate cellular protection and systemic molecular adaptation. However, hypoxia and heat differ in several aspects, including induction modalities, monitoring methods, metrics used to assess physiological strain and substantial intra- and inter-individual variability. Together, these differences challenge direct comparison and/or combination. Therefore, this current opinion highlights research gaps by critically presenting the common and distinct physiological responses and adaptations to hypoxia and heat exposure. It also emphasises the importance of monitoring internal physiological strain rather than external environmental stress to better account for individual variability. Finally, we propose future research perspectives to address current methodological challenges.

## Key Points

**Table:** 

Despite their shared applications in performance and clinical contexts, hypoxia and heat employ markedly different induction methods, monitoring approaches and systemic physiological responses.
Influenced by numerous intra- and inter-individual confounding factors, future research should prioritise quantifying the internal physiological strains rather than the external environmental stress.
Developing a unified environmental stressor dose index is essential for accurately comparing and/or combining hypoxia and heat.

## Introduction

Exposure to hypoxia or heat have become key strategies in both sports’ performance [1–3] and health contexts [4, 5]. Each environmental stressor transiently disturbs homeostasis, eliciting cardiovascular, metabolic and cellular strain that ultimately promotes physiological adaptations [6]. At the molecular level, they converge on shared signalling pathways, notably through the stabilisation of hypoxia-inducible factor-1 alpha (HIF-1α) and the activation of heat shock proteins (HSPs) [7, 8], supporting the concept of cross-tolerance and cross-adaptation [8, 9].

On the basis of these similarities, recent research has explored combined hypoxia and heat exposures applied (i) simultaneously, where both environmental stressors are imposed at the same time (e.g. repeated-sprint training in a hypoxic and hot environment) [10, 11]; (ii) concurrently, where hypoxia and heat are applied within the same day or training block but in distinct sessions (e.g. overnight hypoxic exposure followed by daytime heat training) [12, 13]; and (iii) sequentially, where acclima(tisa)tion to either hypoxia or heat precedes exposure to the other one over several days [14, 15]. A detailed conceptual distinction between these modalities is provided by Girard et al. [16]. Other studies have attempted to directly compare the effectiveness of hypoxia versus heat in eliciting physiological adaptations, for example, in the context of endurance performance [2, 17, 18]. Nevertheless, while these comparisons are often based on literature-recommended protocols for each environmental stressor, the rationale for equating a hypoxic protocol with a heat protocol is generally lacking. Therefore, the quantification of environmental stressor magnitude (i.e. hypoxic versus thermic dose) is essential, yet many issues remain unresolved (e.g. which real-time indices best capture environmental stressor intensity). As Brocherie et al. [7] have highlighted, direct comparisons are highly challenging.

The aim of this current opinion is to examine hypoxic and heat stressors, emphasising both their converging and diverging characteristics, and the key factors that influence their physiological responses, adaptations and interpretation. By synthesising current knowledge on these environmental stressor characteristics, application, dose and individual variability, a conceptual framework is provided to guide researchers and practitioners in considering how hypoxia and heat might be compared, combined or personalised to accelerate and optimise physiological responses and consecutive adaptation. This framework also clarifies conceptual limitations and underexplored areas within the current literature.

## Defining, Inducing, and Quantifying Physiological Strain: Insights from Hypoxia and Heat Stressors

To understand the physiological effect of hypoxia and heat, it is first essential to define the nature of each environmental stressor, outline how they can be applied and describe how their intensity can be quantified. In this context, it is important to distinguish load, stress, strain and dose. Load is defined as the external challenge imposed, stress as the resulting physiological perturbation, strain as the individual response and dose as the cumulative exposure over time.

Table 1 summarises the key features of hypoxic and heat stressors, including their definitions, systemic and local induction methods, approaches for real-time monitoring and strategies for dose quantification. It provides a structured overview to support comparison of these environmental stressors and interpretation of their physiological effects.

**Table 1:** Hypoxia and heat characteristics, monitoring and dose quantification

	Hypoxia	Ref	Heat	Ref
Nature of environmental stress	**Definition** Hypoxia reduces the oxygen availability in human tissues. It can be applied either systemically or locally The physiological responses triggered by hypoxia are detailed in Table 2	[19]	**Definition** Heat increases the temperature of human tissues. It can be applied either systemically or locally The physiological responses elicited by heat are outlined in Table 2	[20]
Induction techniques	**Systemic exposure** HH: real altitude (acclimatisation) or simulated altitude in hypobaric chamber (acclimation).* NH: reduced FiO₂ using chamber, tent, hypoxic mask, voluntary hypoventilation and apnoea (acclimation)	[21, 22]	**Systemic exposure** Natural hot environments (acclimatisation) or climatic chambers, sauna (dry or wet), HWI, heat suit (acclimation)	[14, 27–30]
		[23–26]		
	**Localised exposure** IPC, BFR	[31, 32]	**Localised exposure** Diathermy, infrared lamps, ultrasound, heat-generating pads, heat- and steam-generating plates	[33–37]
Real-time monitoring	**Physiological measurement; devices** - SpO₂; oximeter - O₂Hb and MbO₂, HHb and Mb, total chromophore concentration ([tHb + Mb]), TSI or SmO₂ or TOI (O₂Hb + MbO₂)/(O₂Hb + HHb + MbO₂ + Mb) × 100); NIRS - HR and HRV; chest strap HR monitor or ≥ 3-lead ECG - Ventilatory responses (minute ventilation, breathing frequency, V̇O_2_, V̇CO_2_, RER/RQ, metabolic heat production); gas analyser	[38] [39] [22, 40]	**Physiological measurement; devices** - *T*_rectal_ or *T*_core_; thermistor probe (rectal) or ingestible thermometer capsule - *T*_muscle_; implantable thermistor probe (e.g. at a depth of 2.5 cm) - *T*_skin_; thermistor probe, temperature loggers (iButton, thermocouple) - Heat flux; heat flow sensors - Sweat rate and sweat [Na⁺]; sweat capsules or sweat sensors - HR; chest strap HR monitor or ≥ 3-lead ECG - Ventilatory responses (minute ventilation, breathing frequency, V̇O_2_, V̇CO_2_, RER/RQ, metabolic heat production); gas analyser	[41] [42] [43] [44] [45] [46]
	**Perceptual measurements** - 2018 Lake Louise AMS score, Hackett clinical score, AMS-cerebral score - Environmental symptoms questionnaire - Well-being questionnaire - TS, TC - Perceived thirst scale - RPEs, ROF - Sleep questionnaire (Pittsburgh sleep quality index, Athen’s insomnia scale)	[47–49] [50] [24] [51]	**Perceptual measurements** - Environmental symptoms questionnaire - Wellbeing questionnaire - TS, TC, skin wetness - Perceived thirst scale - RPEs, ROF - PeSI: 5 × ([TS − 1]/4) + 5 × (PE/10), expressed in a score out of 10	[52] [53] [54] [55]
	Cognition measurements** - Attention & processing speed (reaction time (simple and choice), trail making task), - Working memory (memory search, corsi block tapping), - Declarative memory (auditory-verbal learning), - Executive function (Go/No-go task)	[56] [57, 58] [59] [60] [61]	Cognition measurements** - Attention and processing speed (vigilance (psychomotor, visual, auditory), trail making task) - Working memory (digit span and spatial span, N-back test) - Declarative memory (word list recall) - Executive function (Stroop task)	[56] [62, 63] [64] [65] [66]
Dose quantification	**External (stress)** - Hours of exposure at a given altitude - Kilometres-hours (altitude × duration), expressed in km h	[67]	**External (stress)** - AHI: weighted sum of the Humidex over 72 h (see article) - TSD: sum of hours during which the UTCI index remains in a specific category over a day (see article) **-** HSE: (UTCI − 26) × exposure duration (if UTCI > 26 °C)), expressed in °C h⁻^1^	[68] [69] [70]
	**Internal (strain)** - Saturation-hours (SpO₂ × duration), expressed in % h - Hypoxic burden, expressed as relative changes (% min h) compared with pre-desaturation baselines	[38] [71]	**Internal (strain)** - PSI: (5(T_r_t – T_r_0) × (39.5—T_r_0) – 1 + 5(HRt – HR0) × (180 − HR0) – 1), expressed in a score out of 10*** - CHSI: accumulation of thermal load (*T*_core_) and circulatory load (HR) over time. This is a dimensionless index: the higher the value, the greater the heat strain	[72] [73]
	**Internal/external ratio** - SpO_2_ / FiO_2_ ratio - PaO_2_ / FiO_2_ ratio	[74]	**Internal/external ratio** NA	

*AHI* accumulated heat stress index*, AMS* acute mountain sickness, *BFR* blood flow restriction, *CHSI* cumulative heat strain index, *ECG* electrocardiogram*, FiO₂* fraction of inspired oxygen, *HH* hypobaric hypoxia, *HR* heart rate, *HRV* heart rate variability, *HSE* heat stress exposure, *HWI* hot-water immersion, *IPC* ischaemic preconditioning, *Mb* deoxymyoglobin, *MbO₂* oxymyoglobin, *NA* not available, *Na⁺* sodium*, NIRS* near-infrared spectroscopy, *O*_*2*_*Hb* oxyhaemoglobin, *PaO*_*2*_ arterial partial pressure of oxygen, *PE:* perceived exertion score (0–10), *PeSI* perceptual strain index, *PSI* physiological strain index, *RER/RQ* respiratory exchange ratio/respiratory quotient*, RPE* rating of perceived exertion, *ROF* rating of fatigue, *SmO*_*2*_ muscle oxygen saturation, *TC* thermal comfort, *T*_*core*_ core temperature, *T*_*muscle*_ muscle temperature, *TOI* tissue oxygenation index, *T*_*rectal*_ rectal temperature, *T*_*skin*_ skin temperature, *TS* thermal sensation score (1–5), *TSD* thermal stress index, *TSI* tissue saturation index, *SpO*_*2*_ pulsed oxygen saturation, *UTCI* universal thermal climate index, *V̇CO*_*2*_ carbon dioxide production, *V̇O*_*2*_ oxygen consumption

^*^Acclimation occurs in an artificial environment, whereas acclimatisation occurs in a natural one

^**^Numerous cognitive subtests can be used to assess different aspects of cognition. For a complete list, see the cited reference. Here, only one or two examples of tests from each domain are presented

^***^T_rectal_ t: *T*_rectal_ at time *t*. *T*_rectal_0: *T*_rectal_ at baseline. HRt: HR at time *t*. HR0: HR baseline

## Physiological Responses and Adaptation to Hypoxia and Heat

Table 2 summarises the physiological responses and adaptation to hypoxia and heat, distinguishing between whole-body and localised exposures. It illustrates how the environmental-induced physiological strains evolve over time, ranging from minutes to hours (acute), to several days or weeks (repeated or chronic). During this period, ventilatory, cardiovascular, thermoregulatory and cellular adaptations specific to each environmental stressor occur, corresponding to the principle of acclima(tisa)tion. Table 2 also underscores the benefits of combining these environmental exposures with training, as well as their potential side effects. Overall, Table 2 highlights both the common and distinct mechanisms through which these environmental stressors elicit physiological responses and adaptation.

**Table 2:** Overview of physiological responses and adaptation to hypoxia and heat, including acclima(tisa)tion, training and side effects

	Hypoxia	Ref	Heat	Ref
Responses and adaptation	**1. Whole-body hypoxic exposure** **Acute (minutes–hours)** - ↓ PaO₂ and PaCO₂ → ↑ HVR, ↑ minute ventilation (responses are near-instant, within seconds) - Hypocapnia → respiratory alkalosis - ↑ Sympathetic drive → ↑ HR and CO - Peripheral vasodilation, pulmonary vasoconstriction (Euler–Liljestrand reflex) - ↑ ROS, HIF-1α, HSPs, glycolysis, VEGF → hormetic antioxidant and anti-inflammatory responses - ↓ aerobic performance and V̇O_2max_, ↑ RER, anaerobic glycolysis, muscle glycogen utilisation and lactate - CNS: ↑ CBF, impaired motor control (↑ central fatigue during severe hypoxia (SaO_2_ ≤ 75%), ↓ cognition functions)	[75] [76] [77, 78] [79–81] [82–85] [56, 86, 87]	**1. Whole-body heat exposure** **Acute (minutes–hours)** - ↑ *T*_core_ and *T*_skin_ (responses require prolonged exposure, > 10–30 min) - ↑ Cutaneous vasodilation and sweating; blood flow redistributed to skin - ↑ Sympathetic activity → ↑ HR and cardiac contractility to maintain CO - ↓ Splanchnic, renal perfusion - ↑ ROS, HSPs, HIF-1α, VEGF, Akt–mTOR, MAPK → hormetic antioxidant and anti-inflammatory responses - ↓ Aerobic performance, V̇O_2max_, ↑ muscle glycogen utilisation, ↓lipid oxidation - CNS: ↓ CBF, ↑ CMRO_2_, impaired motor control (↑ central fatigue and ↓ cognition functions)	[88] [89] [90, 91] [92–94] [95–97] [56, 98]
	**Repeated (days–weeks)** - ↑ Ventilation and arterial oxygen saturation (SaO₂); renal compensation of alkalosis → restored pH - ↓ Plasma volume → ↑ haemoglobin concentration ([Hb]); ↑ erythropoietin (EPO) → ↑ red cell mass and oxygen (O₂)-carrying capacity, - ↓ Maximal HR and CO; ↑ pulmonary artery pressure - ↑ O₂ utilisation efficiency, metabolic stability and aerobic performance - ↓ Muscle glycogen utilisation, ↑ fat oxidation, ↓ blood lactate and ↑ H⁺/HCO₃^−^/lactate transport - CNS: ↓ central fatigue (↑ brain oxygen delivery and corticospinal excitability)	[99, 100] [101, 102] [87]	**Repeated (days–weeks)** - ↑ Sweat rate and efficiency, ↓ sweating threshold; ↓ electrolyte concentration in sweat - ↑ Cutaneous blood flow, ↓ *T*_core_ threshold for vasodilation, ↑ microvascular sensitivity - ↑ PV → ↑ CV stability and heat capacity; ↑ total body water - ↓ HR, ↑ stroke volume and sustained CO - ↑ HSPs (especially HSP72) → cytoprotection - ↓ *T*_core_ and *T*_skin_, ↑ aerobic performance - ↓ Muscle glycogen utilisation, ↑ fat oxidation, ↓ lactate accumulation, ↓ metabolic rate - CNS: Less affected due to thermoregulatory adaptation	[29, 103] [102, 104]
	**2. Localised hypoxic exposure** **Acute (minutes–hours)** - ↓ Local oxygenation and perfusion - ↑ NO → vasodilation and reactive hyperaemia - Cellular stress (↑ HSP70), ↓ inflammation and apoptosis; ↑ cytoprotection - ↑ Angiogenic gene expression	[105] [106] [81]	**2. Localised heat exposure** **Acute (minutes–hours)** - ↑ *T*_skin_ and *T*_muscle_ - ↑ Local blood perfusion and arterial shear stress, and ↑NO-mediated vasodilation - Cellular stress (↑ HSP27, HSP70, HSP90), ↓ inflammation and apoptosis, ↑ cytoprotection - ↑ Angiogenic gene expression	[31] [107, 108] [46, 109] [46, 110]
	**Repeated (days–weeks)** - ↑ Angiogenesis and oxidative capacity - ↑ Muscle metabolic efficiency, - ↓ Muscle atrophy and strength loss (↑ metabolites, ↓ MuRF1)	[111, 112] [113] [114–116]	**Repeated (days–weeks)** - ↑ Angiogenesis and oxidative capacity - ↓ Muscle atrophy and strength loss (↑ PGC-1α and HSP = ↓ FoxO3 and ↑ Akt/mTOR), - ↑ Muscle hypertrophy and strength (↑ satellite cells, remodelling of the cytoskeleton, ↑ mitochondrial enzyme content & activity, ↑ Akt/mTOR) - ↑ Insulin sensitivity	[117] [118] [119–121] [122]
Acclimation / acclimatisation	**Objective** - ↓ Maladaptive responses (e.g. AMS, HAPE) - ↑ Hypoxia tolerance - ↑ Cognitive and physical performance at altitude	[3]	**Objective** - ↓ Exertional heat illness (e.g. heat stroke) - ↑ Thermal comfort - ↑ Cognitive and physical performance in hot environments	[29]
	**Strategy** - Pre-acclima(tisa)tion: before an ascent (e.g. Everest) - Acclima(tisa)tion: 2–4 weeks, to maintain performance at altitude and/or ↑ aerobic performance at sea level - Re-ascent: restore acclimatisation faster	[123, 124] [3] [125]	**Strategy** - Acclima(tisa)tion: condensed, 1–2 weeks (short-, mid- or long-term), to compete in hot weather conditions (e.g. Tokyo 2020 Olympic Games)* - Re-acclima(tisa)tion: restore adaptations faster, fewer sessions required	[15, 126] [127]
	**Decay of adaptations** - ~ 1-week post-exposure: ~ 50% ↓ Hb_mass_ + fast decay of HR - ~ 2-week post-exposure: Hb_mass_ and ventilation return to baseline	[128]	**Decay of adaptations** - ~ 2-week post-exposure: Hb_mass_, sweat rate, heat tolerance return to baseline - ~ 2.5% daily loss (HR, *T*_core_)	[127, 129]
Combined with training	**Endurance training** Whole-body hypoxic exposure, applied outside of training sessions (e.g. LHTL method) or localised hypoxic exposure, applied during training sessions - ↑ Hb_mass_, ↑ V̇O₂_max_, ↑ buffering capacity ↑ economy of motion **-** ↑ Aerobic performance at sea level	[3, 130, 131]	**Endurance training** Whole-body heat exposure, applied during or outside of training sessions (e.g. HWI) - ↑ Hb_mass_, ↑ V̇O₂_max_, ↑ time-trial - ↑ Aerobic performance at sea level	[132–136]
	**High-intensity intermittent exercise** Whole-body or localised hypoxic exposure applied during training: - submaximal HIIT, IHT: ↑ speed and anaerobic power - RSHyp: ↑ muscle perfusion, glycolysis, energy replenishment (phosphocreatine, glycogen) and metabolite clearance, ↑ repeated sprint performance	[137, 138] [139]	**High-intensity intermittent exercise** Whole-body heat exposure, applied during training: **-** RSHot: ↓ *T*_core_ and *T*_skin_ during exercise, ↑ sweat rate, larger ↑ shuttle running distance (but not significant difference compared with the control group)**	[140]
	**Strength training** Whole-body (RTH) or localised (BFR) hypoxic exposure, applied during training: - ↑ Strength gains - ↑ Muscle hypertrophy - ↑ Fast-twitch recruitment - ↑ Angiogenic signalling	[141–144]	**Strength training** Whole-body or localised heat exposure, applied pre-, during or post-training: - ↑ Neuromuscular activity (acute and chronic) - ↑ Long-term strength gains - ↑ Muscle hypertrophy - ↑ Angiogenic signalling	[35, 145–147]
	**Training recovery** Localised hypoxic exposure, applied pre- or post- exercise: - ↓ Muscle damage, ↓ inflammation	[148]	**Training recovery** Whole-body or localised heat exposure, applied pre- or post- exercise: - ↓ Muscle damage, ↓ inflammation - ↑ Muscle glycogen resynthesis - ↓ Fatigue, ↑ muscle function recovery	[148, 149]
Sides effects	**Whole-body hypoxic exposure** - Disturbed sleep (periodic breathing, apnoea-hypopnoea, phase quality and duration: HH > NH) - Hypertension (prolonged exposure) - AMS, HAPE, HACE - ↓ Immune function - Dehydration - ↓ Cognitive functions (↓attention and processing speed from 2400 m, ↓ working memory > 3600 m, declarative memory stable until ~ 5000 m, ↓ executive functions)	[150] [151] [152] [56]	**Whole-body heat exposure** - Disturbed sleep (phase quality, wakefulness) - ↑ Risk of syncope and orthostatic intolerance - ↑ Blood–brain barrier permeability (if hyperthermia > 40 °C) - ↑ Risk of hyponatraemia - Exertional heat illnesses (heat exhaustion, heat injury, heat stroke) - Dehydration - ↓ Cognitive functions (↓ attention and processing speed affected, ↓ working memory if ↑ *T*_core_ + 1.5 °C, ↓ executive functions on complex tasks, = declarative memory)	[153] [98] [154] [56]
	**Localised hypoxic exposure** - Common side effects: pain, discomfort and cardiac stress - Rare but serious effects: nerve or ischaemic damage, bruising, dizziness, thrombosis and rhabdomyolysis	[155, 156]	**Localised heat exposure** - Redness, superficial burn, or discomfort	[157, 158]

*Akt–mTOR* protein kinase B–mechanistic target of rapamycin pathway, *AMS* acute mountain sickness, *BFR* blood flow restriction, *CBF* cerebral blood flow, *CMRO*_*2*_ cerebral metabolic rate of oxygen, *CNS* central nervous system, *CO* cardiac output, *EPO* erythropoietin, *FiO₂* fraction of inspired oxygen, *FoxO3* Forkhead box protein O3, *HACE* high-altitude cerebral oedema, *HAPE* high-altitude pulmonary oedema, *Hb* haemoglobin, *Hb*_*mass*_ haemoglobin mass, *HH* hypobaric hypoxia, *HIF-1α* hypoxia-inducible factor 1-alpha, *HR* heart rate, *HSPs* heat shock proteins, *HVR* hypoxic ventilatory response, *LHTL* living high-training low, *MAPK* mitogen-activated protein kinase, *MuRF1* muscle RING-finger protein 1, *NH* normobaric hypoxia, *NO* nitric oxide, *O*_*2*_ oxygen, *O*_*2*_*Hb* oxyhaemoglobin, *O*_*2*_* utilisation* oxygen utilisation efficiency, *PaCO*_*2*_ partial pressure of carbon dioxide in arterial blood, *PaO*_*2*_ arterial partial pressure of oxygen, *PGC-1α* peroxisome proliferator-activated receptor gamma coactivator 1-alpha, *PV* plasma volume, *RSHyp* repeated sprint in hypoxia, *RSHot* repeated sprint in heat, *ROS* reactive oxygen species, *RTH* resistance training hypoxia, *SaO*_*2*_ arterial oxygen saturation, *T*_*core*_ core temperature, *T*_*muscle*_ muscle temperature, *TOI* tissue oxygenation index, *T*_*skin*_ skin temperature, *TSI* tissue saturation index, *VEGF* vascular endothelial growth factor, *V̇O*_*2max*_ maximal oxygen uptake

^*^In contrast to hypoxic exposure, the term pre-acclima(tisa)tion is not typically employed in the context of heat interventions

^**^Only if the pre-exercise exposure duration is sufficiently long, given that the kinetics of physiological responses to heat are slower than those to hypoxia

Note: the findings presented in this Table are not unanimous, and some variability exists between studies. As such, there is a tendency towards reporting only positive outcomes while not all research supports all these claims

Furthermore, it should be noted that the effects listed in Table 2 are not consistently observed across all studies (e.g. increase in Hb_mass_) and some outcomes may vary depending on experimental conditions and participant characteristics [2]. Therefore, these findings should be interpreted with caution.

## Hypoxia and Heat Exposure: Comparative Applications and Modulating Factors

**Fig. 1:**
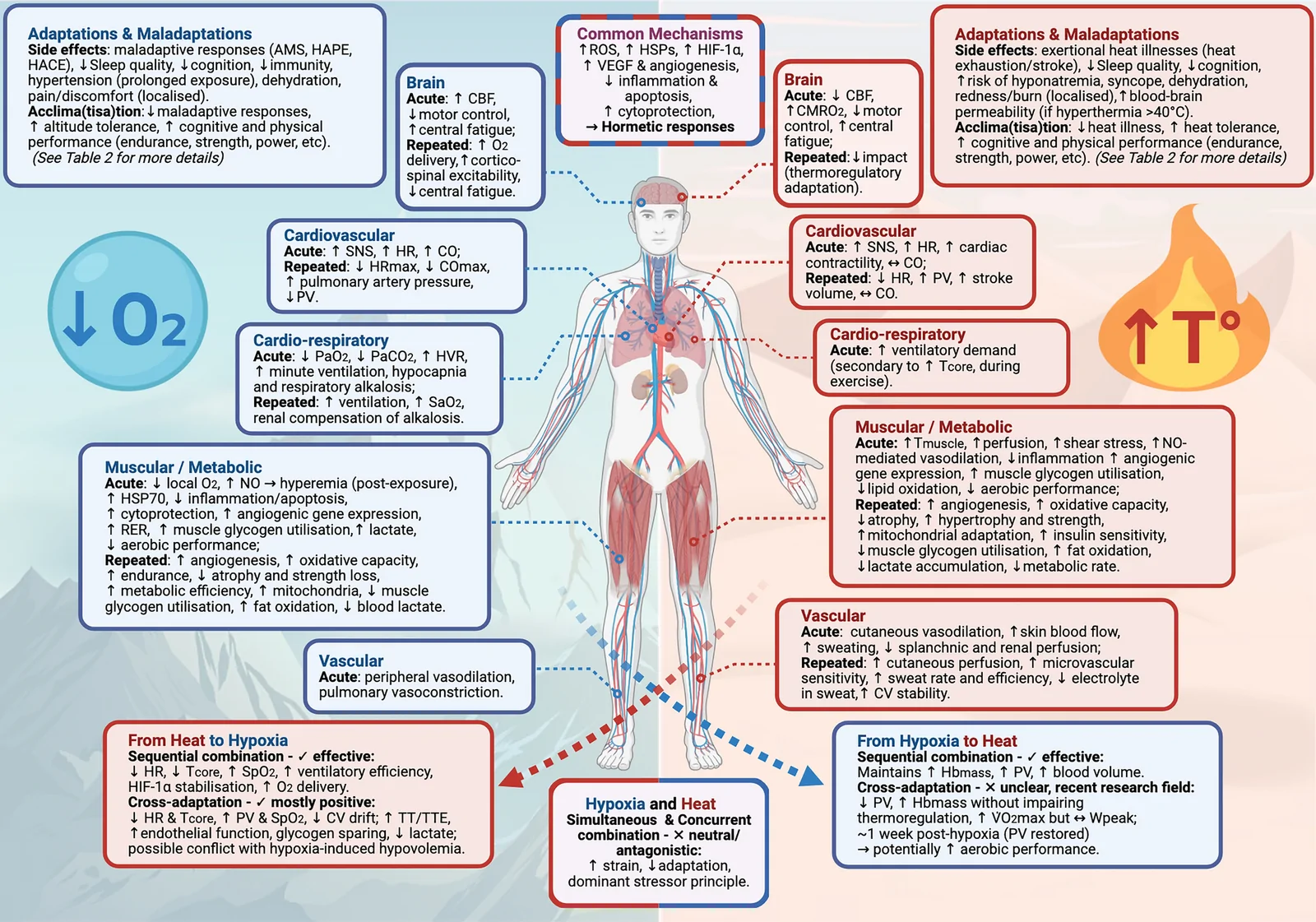
Integrated physiological responses, adaptations and interactions between hypoxia and heat. The top central box shows responses common to both hypoxia and heat, while the bottom box represents combined responses. The blue box (on the left) indicates physiological responses to hypoxia. The red box (on the right) indicates responses to heat. Dotted coloured arrows show the transfer of adaptations between hypoxia and heat, reflecting cross-adaptive or sequential effects in each direction (red box in the hypoxia panel and vice versa). In the boxes: arrows indicate direction of change (↑ = increase, ↓ = decrease), X = not effective, ✓ = effective. *AMS* acute mountain sickness, *CBF* cerebral blood flow, *CMRO*_*2*_ cerebral metabolic rate of oxygen*, CO* cardiac output, *CV* cardiovascular, *HACE* high-altitude cerebral oedema, *HAPE* high-altitude pulmonary oedema, *Hb*_*mass*_ haemoglobin mass, *HIF-1a* hypoxia-inducible factor 1-alpha, *HR* heart rate, *HSP* heat shock proteins, *HVR* hypoxic ventilatory response, *NO* nitric oxide, *O*_*2*_ oxygen, *PaCO*_*2*_ partial pressure of carbon dioxide, *PaO*_*2*_ partial pressure of oxygen, *PV* plasma volume, *RER* respiratory exchange ratio, *ROS* reactive oxygen species, *SaO*_*2*_ arterial oxygen saturation, *SNS* sympathetic nervous system, *SpO*_*2*_ pulsed oxygen saturation, *T°* temperature, *T*_*core*_ core temperature, *T*_*muscle*_ muscle temperature, *TT* time trial, *TTE* time to exhaustion, *VEGF* vascular endothelial growth factor, *V̇O*_*2max*_ maximal oxygen consumption, *W*_*peak*_ peak power. Figure created with BioRender (free trial version)

While Table 2 and Fig. 1 describe the physiological responses and adaptations elicited by hypoxic and heat exposures, they are not uniform across individuals or contexts. Indeed, the same environmental stressor may elicit markedly different levels of physiological strain depending on how, when and in whom it is applied. Consequently, Table 3 embraces a complementary perspective by summarising the main performance and clinical applications of hypoxia and heat exposure, and by identifying major intra- and inter-individual factors that modulate the degree of physiological strain and adaptive outcomes. This could help to interpret the heterogeneity of findings reported in the literature and clarifies why comparable interventions can lead to divergent responses.

**Table 3:** Applications of hypoxia and heat exposures and key modulators of physiological responses and adaptations

	Hypoxia	Ref	Heat	Ref
Applications	**1. Performance** - ↑ Adaptations to endurance, strength, and HIIT, maintain performance at altitude, and ↑ muscle recovery (see Table 2)		**1. Performance** - ↑ Adaptations to endurance, strength and potentially HIIT, maintain performance in warm conditions, and ↑muscle recovery (see Table 2)	
	- Main exposure methods: LHTH, LHTL, LHTLi, LHTLH, LHTLHi, LLTH (IHE, IHT, IHIT, RSHyp, RTH)	[3]	- Main exposure methods: passive exposure, applied either before or after exercise (either whole-body or localised), and active exposure, applied during training (see Table 2)**	
	**2. Clinical** - Metabolic health: ↓ obesity, ↓ diabetes, ↑ metabolic rate, ↑ fatty acid utilisation	[159, 160]	**2. Clinical** - Metabolic health: ↓ obesity, ↓ diabetes (transient), ↑ metabolism	[161–163]
	- Musculoskeletal health: ↓ atrophy, ↑ rehabilitation, ↑ muscle recovery and regeneration, ↑ bone healing	[93, 94, 143, 144]	- Musculoskeletal health: ↓ atrophy, ↑ rehabilitation, ↑ muscle recovery and regeneration, ↑ bone healing	[41, 118, 119, 166]
	- Cardiovascular: ↓ hypertension, ↓ stroke risk, ↑endothelial and vascular function,	[167–170]	- Cardiovascular: ↓ hypertension, ↓ stroke risk, ↑ cardiovascular health	[171–173]
	- Respiratory: ↑ respiratory function, ↓ COPD symptoms	[174]	- Respiratory: ↓ COPD, asthma, chronic bronchitis symptoms	[175, 176]
	- Neurological and mental health: ↓ cognitive decline, ↓ dementia and Alzheimer’s risk, ↓ anxiety and depression	[177–180]	- Neurological and mental health: ↓ cognitive decline, ↓ dementia and Alzheimer’s risk, ↓ mild depression	[181, 182]
	- Oncology: ↓ cancer (e.g. colorectal, breast)	[183]	- Oncology: adjunct in cancer therapy	[184]
	- Pain: NA		- Pain: ↓ chronic pain	[185, 186]
Confounding factors	**1. Intra-individual** - Acclima(tisa)tion status and exercise/training intensity: ↑or↓ physiological strain* (see Table 2)		**1. Intra-individual** - Acclima(tisa)tion status and exercise/training intensity: ↑or↓ physiological strain (see Table 2)	
	- Nutrition: iron status, carbohydrate intake, hydration antioxidants	[187]	- Nutrition: hydration	[95]
	- Circadian rhythm: ↑ HVR and ↓ vLTF in morning compared with evening	[188]	- Circadian rhythm: sweating threshold and vasodilation: ↑ 16:00–20:00, ↓ 00:00–04:00	[189]
	- Sleep deprivation/fatigue: ↓ respiratory response, ↑ HR and BP, ↑ inflammation, ↓ cognitive function	[190]	- Sleep deprivation/fatigue: ↓ skin blood flow and sweating, ↑ sensitivity to heat illness	[53, 191, 192]
	- Menstrual cycle: no effect	[193]	- Menstrual cycle: luteal phase ↑ *T*_core_, delayed sweating, ↑ HR, ↑ min. ventilation, ↑ RPE, ↓ exercise tolerance	[194, 195]
	**2. Inter-individual** - Demographics: age (ageing = ↑ ventilatory, ↓ cardiac and hormonal responses); sex (female/male: ↑ respiratory, ↑cardiovascular, ↓ or = haematological, ↑↓ muscular, ↑autonomic responses)	[196–198]	**2. Inter-individual** - Demographics: age (ageing = ↓ skin blood flow, ↓ sweating, ↓ protective effect of heat therapy); sex (female/male: ↓ sweating, ↓ cardiac output and cutaneous blood flow, ↓ heat production)	[199–202]
	- Training: high-level/endurance-trained = lower HVR, stronger cardiorespiratory coupling	[203]	- Training: high-level/endurance-trained = better heat-loss, faster/longer heat acclima(tisa)tion	[204, 205]
	- Birthplace: highlanders versus lowlanders (↑ lung volumes and diffusion, ↓ pulmonary vasoconstriction, haemoglobin: ↑ in Andeans, ↑ ventilation [HVR])	[206]	- Birthplace: natives of hot climates versus temperate (↑ surface-to-mass ratio, ↓ sweating rate, ↓ skin blood flow ↑ metabolic efficiency (↑ fat utilisation, movement efficiency)	[206]
	- Genetics: high interindividual variability in HVR, SpO₂ and chemosensitivity within a healthy and homogeneous group; ↑ variability in clinical populations	[207–209]	- Genetics: high variability in thermoregulation, cardiovascular, sweating responses within a healthy and homogeneous group; ↑ variability in clinical populations	[44, 204]
	- Morphology: larger lungs (highlanders)	[206]	- Morphology: body size (large = ↑ sudomotor adaptation, small = ↑ haematological adaptation)	[210]
	- Obesity: ↓ lung volume, faster SpO₂ decline	[211]	- Obesity: ↑ *T*_core_, BP, sweating (↑ resting metabolic rate, ↑ sympathetic activation, ↑ adipocytokines)	[212, 213]
	- Muscle fibre type: equal or greater hypertrophy in slow fibres compared with fast fibres (with BFR)	[214]	- Muscle fibre type: fast fibres respond better to heat (↑ HSPs) than slow fibres (animal studies)	[201, 215]

*BFR* blood flow restriction*, BP* blood pressure, *COPD* chronic obstructive pulmonary disease, *HIIT* high-intensity interval training, *HSPs* heat shock proteins, *HVR* hypoxic ventilatory response, *HR* heart rate, *IHE* intermittent hypoxic exposure, *IHIT* intermittent hypoxic exposure during interval training, *IHT* intermittent hypoxic training, *LHTH* live high–train high, *LHTL* live high–train low, *LHTLH* live high–train low and high, *LHTLHi* live high–train low and high (interspersed), *LHTLi* live high–train low (interspersed), *LLTH* live low–train high, *NA* not available, *RPE* rating of perceived exertion, *RSHyp* repeated sprint in hypoxia, *RTH* resistance training hypoxia, *SaO₂* arterial oxygen saturation, *SpO₂* pulsed oxygen saturation, *T*_*core*_ core temperature, *vLTF* ventilatory long-term facilitation

^*^Stress ≠ strain: stress refers to the external or internal demand placed on the body, whereas strain denotes the physiological response or effect resulting from that stress

^**^No protocol systems as structured or clearly defined as LHTL or LLTH for heat exposure

## Combined and Cross-Adaptive Responses to Hypoxia–Heat Exposure

Table 4 presents the physiological and performance outcomes of combined and cross-adaptive hypoxia–heat exposures. It indicates which approaches enhance physiological adaptation, which effects remain uncertain and where simultaneous exposure may be ineffective or even counterproductive.

**Table 4:** Physiological and performance outcomes of combined (simultaneous, concurrent, sequential) and cross-adaptive hypoxia–heat protocols

	**Hypoxia and heat**	Ref
Simultaneous* approach	**Hypoxia + heat:** ✕ neutral/antagonistic - ↑ Acute physiological strain but no additive ergogenic effect - Competing responses: thermoregulatory vasodilation versus hypoxic vasoconstriction - ↑ SpO₂ during RSHyp + hot → undesirable outcome for RSHyp interventions aimed at maximising exercise-induced hypoxaemia - Effect depends on stress severity (“dominant stressor” principle)	[10, 11, 216–218]
Concurrent** approach	**Overnight hypoxia + heat training:** ✕ neutral/unclear - No additional aerobic or performance adaptations - Hypoxia may blunt expected thermoregulatory adaptations	[12, 16, 197, 198]
Sequential*** approach	**Hypoxia → heat:** ✓ effective - Maintains ↑ Hb_mass_, ↑ PV, ↑ blood volume post-altitude - Prevents Hb_mass_ loss	[14]
	**Heat → hypoxia:** ✓ Effective - ↓ HR, ↓ T_core_, ↑ SpO₂, ↑ ventilatory efficiency - HIF-1α stabilisation and enhanced O₂ delivery	[15, 199, 200]
Cross-adaptation (for performance)	**Shared cellular mechanisms:** **-** ↑ HSP70/90 and ↑ HIF-1α activation	[8]
	**Heat acclima(tisa)tion → hypoxic environment:** ✓ mostly positive - Physiological benefits: ↓ submaximal HR, ↓ *T*_core_, ↑ PV, ↑ SpO₂, (at rest and slightly under exercise), ↓ cardiovascular drift - Performance effects: ↑ time-trial, ↑ time-to-exhaustion, glycogen sparing, ↓ lactate, ↑ endothelial integrity and tolerance to hypoxia - Limitations: V̇O_2peak_ and *W*_peak_ minimally affected, conflicting adaptation with hypovolemia in hypoxia	[221–223]
	**Hypoxia acclima(tisa)tion → hot environment:** ✕ unclear, recent research field - Physiological benefits: ↓ PV, ↑ Hb_mass_ does not appear to ↓ efficiency of thermoregulation - Performance effects: ↑ V̇O₂_max_, but unchanged *W*_peak_ - Perspective: optimal windows ~ 1-week post-hypoxic acclima(tisa)tion restore PV and could ↑ aerobic performance	[223, 224]

*Hb*_*mass*_ haemoglobin mass, *HIF-1α* hypoxia-inducible factor-1 alpha, *HR* heart rate, *HSP* heat shock proteins, *O*_*2*_ oxygen, *PV* plasma volume, *RSHyp* repeated sprint in hypoxia, *RSHot* repeated sprint in heat, *SpO*_*2*_ pulsed oxygen saturation, *T*_*core*_ core temperature, *V̇O*_*2max*_ maximal oxygen uptake, *V̇O*_*2peak*_ peak oxygen uptake, *W*_*peak*_ peak power, *✕* unclear, *✓* effective

^*^Simultaneous: both environmental stressors are imposed at the same time

^**^Concurrent: hypoxia and heat are applied within the same day or training block but in distinct sessions

^***^ Sequential: acclima(tisa)tion to either hypoxia or heat precedes exposure to the other one over several days

## Observations, Recommendations and Perspectives

To guide future practice, we highlight key observations that emerge from the current literature before outlining their practical applications.

Observation no. 1: Table 1 highlights large differences in dose quantification. In hypoxia, dose is often estimated using a limited set of oxygenation-related variables (e.g. pulsed oxygen saturation [SpO₂]), thus enabling relatively direct links to be established between external stress and internal strain. In contrast, the heat dose relies on composite indices combining multiple physiological markers (e.g. heart rate (HR), core temperature [*T*_core_]), developed primarily to ensure safety (e.g. physiological strain index [PSI], cumulative heat strain index [CHSI]). Conversely, hypoxia metrics (e.g. kilometres–hours, saturation–hours) were designed to characterise exposure for performance optimisation. Hypoxia research also appears more advanced in formalising dose via internal-to-external ratios, which have no equivalent in the heat-related literature.

Observation no. 2: Table 2 reports the differences in physiological responses to hypoxia and heat. Temporal differences are also evident, ranging from the kinetics of acute responses (faster in hypoxia than in heat) to the duration of acclima(tisa)tion, which is generally shorter for heat than for hypoxia. These differences suggest that hypoxia and heat cannot be compared in terms of adaptive ‘efficacy’ but rather be considered as distinct environmental stressors.

Observation no. 3: Table 3 illustrates a wide range of intra- and inter-individual confounders (e.g. acclima(tisa)tion, nutrition, sleep, menstrual cycle, age, sex, morphology and muscle fibre type) that may exert a significant influence on physiological responses and adaptations. These factors emphasise the importance of individualising exposures.

Observation no. 4: Finally, the findings presented in Table 4 indicate that sequential protocols confer physiological and performance benefits, whereas simultaneous and concurrent approaches appear to yield limited or no additional benefit. Nevertheless, the conclusions of these studies focus mainly on endurance performance, leaving their impact on strength and power training to be explored.

Recommendations: Rather than prescribing a fixed external environmental stressor, the focus should be on targeting the internal physiological responses by defining target zones using reliable physiological markers for both hypoxia (i.e. SpO_2_) and heat (i.e. *T*_core_), monitoring in real time and adjusting environmental exposure as needed. These zones should be adapted to training status, acclima(tisa)tion history and specific objectives such as performance optimisation or health promotion. Nevertheless, practical implementation faces several challenges, including technical and human resource requirements, time constraints and equipment limitations. Therefore, a realistic first step is not to aim for a precise ‘targeting’ of physiological responses, but to systematically monitor and quantify them. This approach underscores the need for caution when directly comparing the benefits of hypoxia versus heat solely on the basis of physiological outcomes.

Perspectives: Hypoxia and heat cannot be matched in magnitude, making direct comparisons solely on the basis of physiological outcomes challenging [7]. Consequently, there is an urgent need to record and monitor psychological and perceptual responses. Therefore, the development of shared psychophysiological or perceptivo-physiological indices could provide a unified measure of systemic strain. To date, no consensus exists regarding specific indices related to environmental stressors. Importantly, these indices should be easy to measure, accessible and preferably non-invasive, ensuring that their use is not restricted by cost, time, available resources or technical expertise. At this stage, these are purely preliminary concepts, highlighting the need to develop a concrete method for directly comparing these environmental stressors. To this end, we propose the term ‘PEARL’ for physio-environmental adaptive response load, for considering a panel of physiological and perceptual markers.

It should be noted that the term PEARL is intended to represent an exploratory framework to reconcile the fundamentally different characteristics of hypoxic and heat stressors with their often convergent physiological and adaptive outcomes. PEARL describes the integrated physiological strain elicited by an environmental stressor’s exposure, arising from the combined activation of multiple systems rather than from the external stress per se. In this view, environmental dose is interpreted through the magnitude of internal adaptive load, allowing hypoxia and heat to be compared, combined and applied within a shared dose–response framework despite distinct underlying mechanisms. At this stage, PEARL is advanced as a heuristic construct rather than a quantified index, with future research needed to define its components, relative contributions and potential operationalisation.

Developing such integrative approaches would enhance our understanding of environmental stressors and improve their application, either individually or in combination (e.g. simultaneous, concurrent, sequential), across various areas of interest.

## Conclusions

Hypoxia and heat share many overlapping physiological pathways that regulate cellular homeostasis, promote cytoprotection and drive adaptive remodelling, all converging to improve sport/physical performance and health. However, differences in induction modalities, monitoring techniques and biological variability currently make any direct comparison premature and unfeasible. Future research should seek to establish a common environmental stressor dose index that would enable rigorous comparisons between hypoxia and heat, while also optimising their application, either individually or in combination, and the resulting physiological adaptations.
